# Psychometric properties of the Arabic version of the intolerance of uncertainty scale: a multinational study

**DOI:** 10.1186/s40359-024-01631-x

**Published:** 2024-03-15

**Authors:** Roni Chaaya, Rabih Hallit, Alvaro Postigo, Diana Malaeb, Fouad Sakr, Mariam Dabbous, Amthal Alhuwailah, Hanaa Ahmed Mohamed Shuwiekh, Sahar Obeid, Feten Fekih-Romdhane, Souheil Hallit

**Affiliations:** 1 School of Arts and Sciences, Social and Education Sciences Department, Lebanese American University, Jbeil, Lebanon; 2 School of Medicine and Medical Sciences, Holy Spirit University of Kaslik, Jounieh, P.O. Box 446, Lebanon; 3 Department of Infectious Disease, Bellevue Medical Center, Mansourieh, Lebanon; 4 Department of Infectious Disease, Notre Dame des Secours University Hospital, Postal code 3, Byblos, Lebanon; 5 Department of Psychology, University of Oviedo, Oviedo, Spain; 6 College of Pharmacy, Gulf Medical University, Ajman, United Arab Emirates; 7 School of Pharmacy, Lebanese International University, Beirut, Lebanon; 8 Department of Psychology, Kuwait University, Kuwait, Kuwait; 9 Department of Psychology, Fayoum University, Fayoum, Egypt; 10 The Tunisian Center of Early Intervention in Psychosis, Department of Psychiatry “Ibn Omrane”, Razi hospital, 2010 Manouba, Tunisia; 11 Faculty of Medicine of Tunis, Tunis El Manar University, Tunis, Tunisia; 12 Applied Science Research Center, Applied Science Private University, Amman, Jordan; 13 Department of Psychology, Badr University of Cairo, Cairo, Egypt

**Keywords:** Intolerance of uncertainty, IUS-12, Prospective anxiety, Inhibitory anxiety, Arabic, Psychometric properties

## Abstract

**Introduction:**

It is common for people to experience anxiety when contemplating their unknown future. Studies have shown that those who tend to worry more about their future are more likely to be intolerant of uncertainty. In order to study the way people from the Middle East tackle uncertainty, a valid and reliable scale is needed. In this context, the present study aimed to examine the psychometric properties of the Arabic version of the 12-item Intolerance of Uncertainty Scale (IUS-12) in a community sample of native Arabic–speaking participants from Lebanon, Egypt, and Kuwait.

**Methods:**

A sample of 2038 university students answered the survey, with a mean age of 22.30 ± 4.15 years and 77.5% females. A Google Form link was dissipated among participants that included some demographic questions, the IUS-12 and the Depression, Anxiety and Stress Scale (DASS-8).

**Results:**

Following the confirmatory factor analysis (CFA), a bi-dimensional model of the Arabic version of the IUS-12 was found. The scale showed an excellent internal reliability for the prospective anxiety (ω = 0.85 / α = 0.85) and inhibitory anxiety (ω = 0.87 / α = 0.87). Additionally, the results illustrated configural, metric, and scalar invariance across genders and countries. Furthermore, Egypt and Lebanon were seen to have statistically significant higher levels of inhibitory anxiety compared to Kuwait, while only Egypt showed statistically significant higher prospective anxiety compared to Kuwait. Finally, higher psychological distress was significantly and positively associated with higher prospective and inhibitory anxiety.

**Conclusion:**

The results of this study provide support for the psychometric reliability and validity of the Arabic version of the IUS-12, allowing for its generalizability and suitability for use among individuals from different Arabic-speaking nations.

## Introduction

When contemplating their futures, individuals may experience feelings of anxiety especially when there is uncertainty revolving around the outcome of anticipated events, a state that is characteristic of worry [1]. While worry is common in the general population, it can be a core characteristic of Generalized Anxiety Disorder (GAD) when it is uncontrollable and disproportionate to the situation [1, 2]. The greater the extent to which people worry or ruminate about the potential occurrence of an adverse event, the higher the levels of anxiety are maintained [3]. In the 1990s, a cognitive model for GAD was postulated from the study of worry [4]. This new cognitive model illustrated four components of GAD, namely: intolerance of uncertainty, poor problem-orientation, cognitive avoidance, and positive beliefs about worry [2]. Intolerance of uncertainty (IU) was studied and found to be a fundamental element involved in worry, with levels of IU being higher in worriers compared to those who do not worry as much [2]. From a cognitive context, individuals with high levels of anxiety tend to interpret possible future events as threatening, preventing them from tolerating the future’s uncertainty or the unknown [5]. In a related context, in the treatment of GAD, it was proven that working on improving one’s tolerance of uncertainty leads to the alleviation of anxiety symptoms [6].

Although there is no single definition for the term “intolerance of uncertainty” [5], it has been conceptualized as a notion whereby negative situations and outcomes are anticipated when the prediction of such events with absolute certainty is not feasible [3]. In other words, an individual is considered intolerant of uncertainty when they have an excessive inclination to deem the occurrence of any future negative event as unacceptable, regardless of how minimal its probability of happening is [1]. The more the uncertainty and ambiguity revolving around the anticipated event, the more intolerant an individual would be [1]. Similarly, greater levels of IU have been found to be inversely proportional to decision-making abilities, coping skills, motivation, and academic performance [7]. Therefore, IU has been regarded by some researchers as a cognitive, emotional, and behavioral response to ambiguity and uncertainty in daily life occurrences [2, 8].

Furthermore, other studies have elaborated on the vulnerability aspect of IU, being correlated with a number of other cognitive vulnerabilities such as rumination, susceptibility to anxiety and fear of negative appraisal [9]. IU has also been found to be inversely related to psychological wellbeing [10]. Additionally, IU is assumed to be accountable for the high comorbidity between anxiety disorders, depressive disorders, and stress [7]. Furthermore, in children, adolescents [11] and adults [12], IU was found to be associated with anxiety.

In order to operationalize IU, a 27-item Intolerance of Uncertainty Scale (IUS-27) was initially developed in French in 1994 to evaluate responses encompassing emotions, cognitions and behaviors in the face of uncertain events, as well as the consequences of being uncertain, and the efforts put to take control over future outcomes [8]. The IUS-27 was seen to have excellent internal consistency (α = 0.91), good test-retest reliability (*r* =.74) and good discriminant and convergent validity [2, 3, 8]. The 27 items of the IUS are scored on a 5-point Likert scale ranging from 1 (*not at all characteristic of* me) to 5 (*entirely characteristic of* me) [3]. It also consists of 5 factors, namely: (1) “unacceptability and avoidance of uncertainty”, (2) “negative social evaluation caused by uncertainty”, (3) “uncertainty-related frustration”, (4) “uncertainty causes stress”, and (5) “uncertainty preventing action” [3]. The scale was then translated into English and a confirmatory factor analysis (CFA) was conducted yielding a scale with a smaller number of items with the absence of the five factors previously seen in the IUS-27 [3]. It was found that the IUS-12 excluded the GAD-specific items of the IUS-27 [7]. Additionally, the IUS-12 consisted of two factors, Prospective Anxiety/IU and Inhibitory Anxiety/IU [3, 5, 7]. The 12-item IUS ensued with excellent internal consistency [3]. Prospective anxiety refers to an individual’s inclination towards minimizing uncertainty by actively seeking out information and predicting future events [7, 13]. This factor signifies the cognitive assessment of future uncertainties, making it the cognitive component of IU [5, 7]. For instance, an item within this IU factor would be “one should always look ahead so as to avoid surprises” [5]. On another hand, inhibitory anxiety, the behavioral component of IU [7], relates to an avoidant reaction to uncertainty [14]. It signifies the inhibition of behaviors in the face of uncertainty and would include items such as “When it’s time to act, uncertainty paralyses me” [5, 14].

The IUS-12 has been translated and validated among different cultures, namely Brazilian [5], Iranian [7] and British [13]. All these studies have found a two-factor structure for the scale similar to the one previously discussed, without having to remove any item [5, 7, 13]. The translated IUS-12 was seen to have strong internal consistency with a Cronbach’s alpha of 0.88 [5] and 0.89 [7] for the scale in general, and for the subscales as well [5, 13]. On another hand, other studies suggested a unidimensional structure for the IUS-12, suggesting the utilization of one total score for IU [13, 15, 16].

### The present study

In this study, the main aim was to explore the psychometric properties of a novel Arabic version of the IUS-12 in a sample of Arabs from Lebanon, Egypt, and Kuwait. The IUS was the scale chosen for this study because it has previously demonstrated excellent psychometric properties [7] and alternative scales that measure IU tend to be longer (such as the Uncertainty Response Scale [17]) or have poor psychometric properties (Intolerance of Ambiguity Scale [18]) [3].

More specifically, this study will help shed light on the way people from the Middle East tackle uncertainty, with previous research pointing out that Arabs in countries such as Libya, Saudi Arabia, Egypt, and Kuwait tend to have higher scores of uncertainty avoidance compared to Americans and British [19]. Therefore, by studying the psychometric properties of the newly translated Arabic version of the IUS-12, a novel instrument will be available for the measurement of IU in Arab countries. It is worthy to note that there is a standard, official, and more formal Arabic language used uniformly across all Arabic-speaking countries known as ‘Fusha’ which is utilized in literary and academic reading and writing [20]. Fusha is used to account for the language barrier between Arabic dialects that are used on a daily basis and that differ from one Arabic-speaking nation from another [20].

Furthermore, Middle Eastern countries stand out as the world’s most politically unstable regions [21]. For instance, in Lebanon, the recurrent occurrence of conflicts and wars throughout Lebanon’s history has instilled a feeling of apprehension and uncertainty in the Lebanese people regarding their present and future [22]. Moreover, the continuous uncertainty experienced by the Lebanese can be attributed to the wars in the neighboring countries, the regular conflicts between the various political parties and religious groups within Lebanon, and the nation’s feeble security as a whole [22]. Similarly, in Egypt, significant instability was characterized by ongoing changes in governmental leaderships, escalating political turmoil [23]. This situation impacted the wellbeing of the population, inducing psychological distress and uncertainty regarding their future prospects [23]. On the other hand, the Gulf region is characterized by political stability [24], and Kuwait, as a member of this region, exemplifies a high-income economy [25]. However, citizens in Kuwait tend to exhibit feelings of uncertainty in novel situations, such as the COVID-19 pandemic [22, 26] With the high levels of uncertainty among the Arabic population in general, it is important to evaluate the psychometric properties of the Arabic version of the IUS-12 among a group of Arab-speaking participants from Lebanon, Egypt and Kuwait.

## Methods

### Procedures

Data collection took place in July 2023 through a Google Form link. Through snowball sampling technique, the research team reached out to university students within their social circle who were asked to share the link with other students that they know. Inclusion criteria required participants to be residents and citizens of Lebanon/Egypt/Kuwait and aged 18 years and above. Excluded were participants not fulfilling these criteria. The Google Form consisted of an introductory paragraph highlighting the study’s aims and ensured participants’ confidentiality and anonymity of their responses. Following their informed consent given digitally, participants voluntarily completed the instruments provided in the questionnaire without any remuneration.

### Measures

#### Demographics. Participants were asked to provide their age and sex

##### Intolerance of uncertainty scale (IUS-12)

The IUS-12, a shortened version of the IUS-27 [3], consists of 12 items that are scored on a Likert scale that ranges from 1 (*Not at all characteristic of me*) to 5 (*Very characteristic of me*) [7]. The IUS-12 includes two subscales of IU, namely prospective anxiety and inhibitory anxiety, with higher scores representing higher levels of anxiety [7].

##### Depression, anxiety, and stress scale (DASS-8)

The DASS-8 is an abridged version of the DASS 21 comprising eight items categorized into three subscales: three items for depression, three items for anxiety and 2 items for stress [27]. A 4-point Likert scale is used to score the items, ranging from 0 (*did not apply to me at all*) to 3 (*applied to me very much or most of the time*) [27]. The total score of the DASS-8 ranges between 0 and 24. The subscale scores for depression and anxiety range between 0 and 9, while that of the stress subscale ranges between 0 and 6 [28] (ω = 0.84 / α = 0.83).

### Analytic strategy

#### Data treatment

The dataset did not include any missing responses. A Confirmatory Factor Analysis (CFA) was used to study the factor structure of the IUS using the data from the total sample via SPSS AMOS v.29 software. The minimum sample size to perform a CFA ranges from 3 to 20 times the number of the scale’s variables [29]. Hence, we deemed a minimum sample size of 36–240 participants necessary to ensure sufficient statistical power. The aim was to evaluate the one- and two-factor models of the scale as documented in previous research studies. Parameter estimates were derived using the maximum likelihood method. Fit indices, including the normed model chi-square (χ²/df), the Steiger-Lind root mean square error of approximation (RMSEA), the Tucker-Lewis Index (TLI) and the comparative fit index (CFI), were calculated. Values ≤ 5 for χ²/df, and ≤ 0.08 for RMSEA, and 0.90 for CFI and TLI indicate good fit of the model to the data [30]. Initial verification did not confirm multivariate normality (Critical ratio > 5; Bollen-Stine *p* =.008); therefore, a non-parametric bootstrapping procedure was conducted.

#### Measurement invariance

To assess gender and country invariance of IUS scores, we conducted multi-group CFA [31] using the total sample. Measurement invariance was evaluated at the configural, metric, and scalar levels [32]. We accepted ΔCFI ≤ 0.010 and ΔRMSEA ≤ 0.015 or ΔSRMR ≤ 0.010 as evidence of invariance [31].

#### Further analyses

Composite reliability was evaluated using McDonald’s ω and Cronbach’s α, with values greater than 0.70 being indicative of adequate composite reliability. Normality of the IUS subscales scores was verified since the skewness and kurtosis values for each item of the scale varied between − 1 and + 1 [33]. For concurrent validity assessment, Pearson test was employed to examine the correlation between IUS scores and DASS-8 scores. Gender-based comparison was conducted using the Student *t* test only if scalar or partial scalar invariance and between countries using the ANOVA test. Post-hoc analysis was done using the Bonferroni test to discern a significant difference between countries taken two by two.

## Results

A total of 2038 university students answered the survey, with a mean age of 22.30 ± 4.15 years and 77.5% females. The details by country are found in Table 1. The description of the IUS items are summarized in Table 2.

**Table 1:** Description of the sample by country

	Total (*n* = 2038)	Egypt (*n* = 674)	Kuwait (*n* = 740)	Lebanon (*n* = 624)
Age (years)	22.30 ± 4.15 [min = 18; max = 40]	20.96 ± 1.95 [min = 18; max = 39]	23.56 ± 5.82 [min = 18; max = 40]	22.27 ± 2.85 [min = 18; max = 39]
Gender				
Males	459 (22.5%)	120 (17.8%)	105 (14.2%)	234 (37.5%)
Females	1579 (77.5%)	554 (82.2%)	635 (85.8%)	390 (62.5%)

**Table 2:** Parametric properties of the Intolerance of Uncertainty Scale’s items in the total sample

	Mean	SD	Median	Skewness	Kurtosis	Alpha if item deleted
IUS 1	2.79	1.15	3.00	0.114	− 0.775	0.90
IUS 2	2.89	1.14	3.00	0.057	− 0.747	0.90
IUS 3	3.01	1.21	3.00	0.005	− 0.937	0.90
IUS 4	2.83	1.4	3.00	0.159	− 0.947	0.90
IUS 5	2.91	1.26	3.00	0.082	-1.023	0.90
IUS 6	2.63	1.19	3.00	0.263	− 0.787	0.89
IUS 7	3.06	1.19	3.00	− 0.030	− 0.881	0.90
IUS 8	2.35	1.18	2.00	0.500	− 0.678	0.90
IUS 9	2.33	1.18	2.00	0.449	− 0.795	0.90
IUS 10	2.73	1.17	3.00	0.196	− 0.802	0.89
IUS 11	2.48	1.17	2.00	0.365	− 0.721	0.89
IUS 12	2.68	1.19	3.00	0.263	− 0.752	0.90
Prospective anxiety	20.10	6.09	20.00	0.085	− 0.430	-
Inhibitory anxiety	12.56	4.74	12.00	0.396	− 0.320	-

### Confirmatory factor analysis

**Fig. 1:**
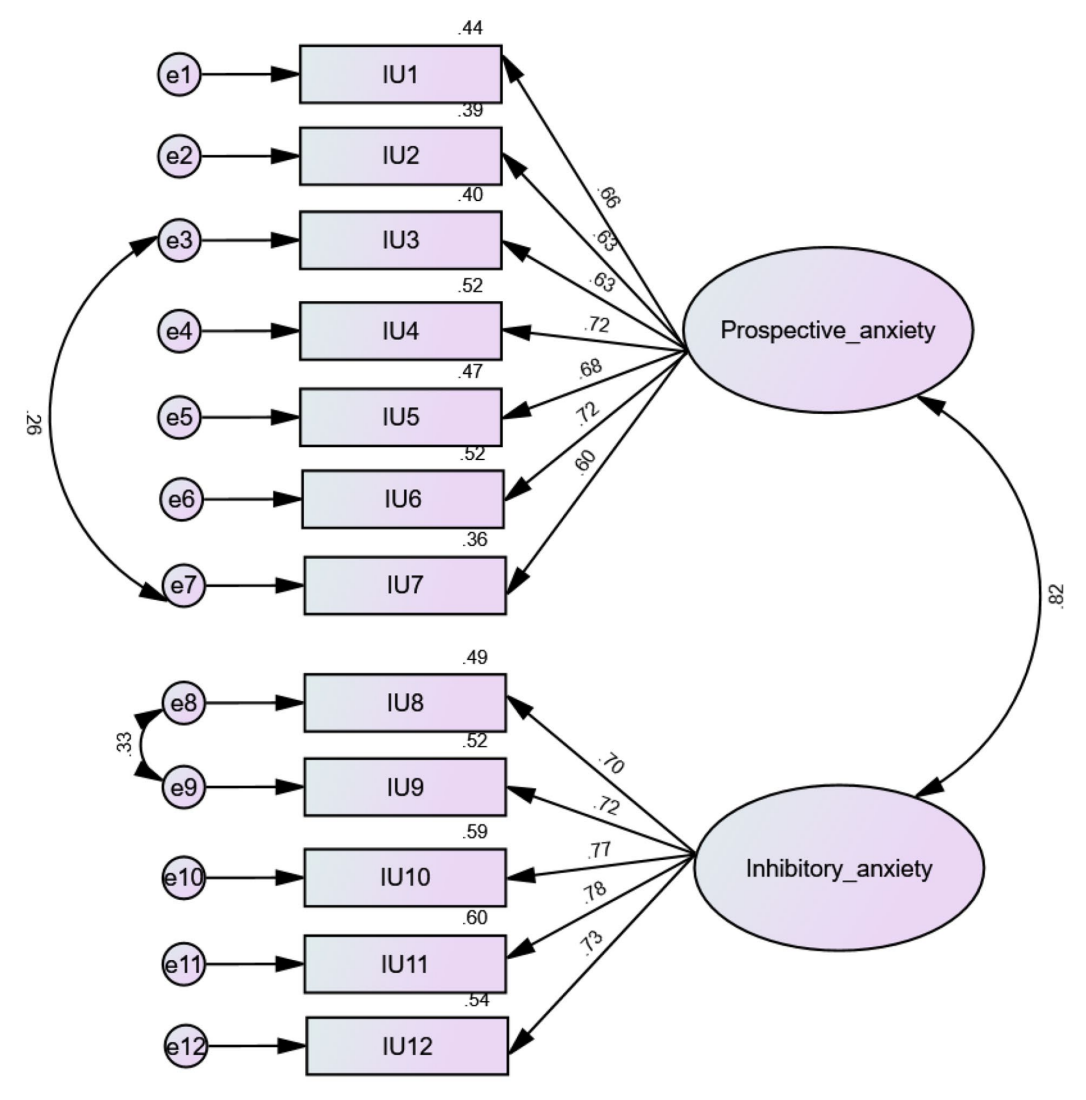
Standardized loading factors of the Intolerance of Uncertainty (IU) scale in the total sample

CFA indicated that fit of the two-factor model of IUS scores was modest: RMSEA = 0.083 (90% CI 0.078, 0.088), SRMR = 0.044, CFI = 0.932, TLI = 0.915. We added a correlation between residuals of items 3–7 and 8–9 since the modification indices were high; the numbers improved as follows: RMSEA = 0.067 (90% CI 0.062, 0.072), SRMR = 0.067, CFI = 0.958, TLI = 0.945. The standardized estimates of factor loadings were all adequate (see Fig. 1). Internal reliability was excellent for the prospective anxiety (ω = 0.85 / α = 0.85) and inhibitory anxiety (ω = 0.87 / α = 0.87).

It is of note that the fit indices of the one-factor model of IUS was poor: RMSEA = 0.115 (90% CI 0.110, 0.120), SRMR = 0.063, CFI = 0.868, TLI = 0.838.

### Measurement invariance

Indices suggested that configural, metric, and scalar invariance was supported across genders and countries (Table 3). No significant difference was found between males and females in terms of prospective anxiety (20.12 ± 6.11 vs. 20.10 ± 6.08, *t*(2036) = 0.052, *p* =.958) and inhibitory anxiety (12.66 ± 4.65 vs. 12.53 ± 4.76, *t*(2036) = 0.516, *p* =.606) scores.

In terms of countries, a higher mean prospective anxiety mean score was found in Egypt (20.46 ± 6.21) compared to Lebanon (20.31 ± 5.74) and Kuwait (19.61 ± 6.23), with a significant difference seen between Egypt and Kuwait only (*p* =.026), F(2,2035) = 3.97, *p* =.019. Finally, a higher mean inhibitory anxiety mean score was found in Lebanon (13.07 ± 4.49) compared to Egypt (12.67 ± 4.95) and Kuwait (12.03 ± 4.70), with a significant difference seen between Egypt and Kuwait (*p* =.035) and between Lebanon and Kuwait (*p* <.001), F(2,2035) = 8.45, *p* <.001.

**Table 3:** Measurement Invariance across gender and country in the total sample

Model	CFI	RMSEA	SRMR	Model Comparison	ΔCFI	ΔRMSEA	ΔSRMR
Model 1: Gender							
Males	0.910	0.099	0.057				
Females	0.935	0.081	0.044				
Configural	0.929	0.060	0.057				
Metric	0.928	0.058	0.059	Configural vs. metric	0.001	0.002	0.002
Scalar	0.928	0.056	0.059	Metric vs. scalar	< 0.001	0.002	< 0.001
Model 2: Country							
Lebanon	0.921	0.098	0.055				
Egypt	0.938	0.083	0.045				
Kuwait	0.920	0.083	0.047				
Configural	0.926	0.051	0.045				
Metric	0.925	0.048	0.048	Configural vs. metric	0.001	0.003	0.003
Scalar	0.920	0.047	0.048	Metric vs. scalar	0.005	0.001	< 0.001

Note. CFI = Comparative fit index; RMSEA = Steiger-Lind root mean square error of approximation; SRMR = Standardised root mean square residual

### Concurrent validity

Higher prospective anxiety was significantly associated with higher total psychological distress (*r* =.27; *p* <.001), depression (*r* =.23; *p* <.001), anxiety (*r* =.23; *p* <.001), and stress (*r* =.25; *p* <.001) in the total sample. Moreover, higher inhibitory anxiety was significantly associated with higher total psychological distress (*r* =.26; *p* <.001), depression (*r* =.25; *p* <.001), anxiety (*r* =.25; *p* <.001), and stress (*r* =.14; *p* <.001).

## Discussion

The main goal of the present study was to evaluate the psychometric properties of the Arabic version of the IUS-12, an efficient tool that aims to quantify their emotions, cognitions, and behaviors in light of uncertainty about their future, among a group of Arab-speaking participants from Lebanon, Egypt and Kuwait. The results demonstrated a two-factor model that presented excellent internal reliability for both the Prospective anxiety and Inhibitory anxiety subscales. This is consistent with the findings of other researchers who supported the two-factor model of the IUS-12, with the prospective anxiety and inhibitory anxiety being the two main factors [3, 5, 7]. This is contrary to the findings of other studies who advocated the use of a unidimensional structure of the IUS-12 [15, 16]. Consequently, the reported findings illustrate the Arabic version of the IUS-12 to be a reliable and valid tool to evaluate IU in an Arab sample.

In regards to the comparison between male and female participants, this study highlighted configural, metric, and scalar invariance across genders. Therefore, in terms of prospective and inhibitory anxiety, no significant difference was reported between the scores of males and females. This is contradictory to the findings that were reported by the Brazilian sample that illustrated significant gender differences on the IUS-12, with females scoring higher means on the IUS-12 total score, prospective anxiety, and inhibitory anxiety [5].

As for the comparison of IUS-12 scores between countries, this study found that, on the prospective anxiety subscale, Egyptians scored higher than the Lebanese and Kuwaitis. On the contrary, Lebanese had the highest mean score on the inhibitory anxiety subscale. One explanation to this finding is the concept that a low socioeconomic status or a decrease in income is associated with greater intolerance of uncertainty, especially inhibitory IU [34]. This might be explained by the fact that Kuwait consists of a high-income economy and is part of the Gulf Cooperation Council (GCC) [25], while Lebanon has a failing economy with more than two-thirds of its citizens living below the poverty line [35]. Similarly, Egypt has been experiencing high inflation and an increase in its poverty line across the years [36].

Finally, psychological distress was seen to be associated with higher levels of prospective and inhibitory anxiety. This is concordant with the results of another study that found that both subscales of the IUS-12 positively correlated with psychological distress [37]. One study conducted on medical students found that inhibitory anxiety tends to be associated with psychological distress [38]. These results can be explained by anxiety and worry being associated with making decisions in light of uncertainty, leading to behavioral and cognitive paralysis in the face of ambiguity out of fear from the consequences of one’s decisions [38]. Some researchers suggest that the perceived threat from the uncertain future is what contributes to distress [39]. In other words, as the subjective extent to which one feels threatened by the uncertainty ahead increases, an increase in the distress felt is experienced, adding proof for the association between uncertainty and psychological distress [39]. In a study conducted during the COVID-19 pandemic, IU was the lead contributor for the variance in psychological distress, with social support being a lead protective factor in alleviating both variables [40].

### Study limitations

Some of the shortcomings present in the current study, which can be worked upon in future research, include the method of recruitment used to gather participants. For instance, the sample gathered does not represent the entire Lebanese, Egyptian or Kuwaiti populations. Other limitations that need to be taken into consideration are the inconsistencies in gender samples, with female participants being more represented than males, especially since other studies have found differences in IU scores between genders [5]. Moreover, this study did not control for all demographic variables, such as academic levels of participant, residence status, economic status, and employment status, which are all factors that could potentially influence the study’s outcomes. This is especially true noting that Kuwait is situated in the Gulf region, which exhibits greater political and economic stability [24, 25] compared to Middle Eastern countries like Lebanon and Egypt [10, 23]. Thus, the generalizability of this study’s findings is impeded by the lack of control over these variables and other variables like the age of participants and their ethnicities. Additionally, since the sample gathered was from the general population, this affects generalizability of the results to clinical samples, i.e. people diagnosed with GAD [7]. Future studies should focus on studying the IUS-12 in a clinical sample to explore the scale’s strength among such a sample [7], while associating IU with different anxiety-related constructs [5]. Finally, self-reporting bias might be an issue when participants fill out a self-reported scale, such as the IUS-12. Future research should focus on controlling for the cultural differences among Arab nations, taking into consideration the socio-political and economic differences of Arab countries. Narrowing the study exclusively to Middle Eastern countries or Gulf nations that share similar cultural, social, political, and economic statuses would be ideal for controlling demographic differences.

## Conclusion

The results of this study offer support for the psychometric soundness of the Arabic adaptation of the IUS-12. This version has been proven efficient for evaluating individuals’ reactions to uncertainty, encompassing emotions, thoughts, and behaviors, as well as their ability to tolerate the ambiguity of the future and the consequences of their decisions in the face of uncertainty. The introduction of the Arabic IUS-12 is intended to simplify the in-depth examination of associations between IU and various psychological variables and sociodemographic factors within a cultural and linguistic framework. It will also facilitate cross-national research partnerships and comparisons involving Arab nations.

## Data Availability

All data generated or analyzed during this study are not publicly available. The dataset supporting the conclusions is available upon request to the corresponding author.
