# Correction to: Psychometric properties of the arabic version of the intolerance of uncertainty scale: a multinational study

**DOI:** 10.1186/s40359-024-01712-x

**Published:** 2024-04-12

**Authors:** Roni Chaaya, Rabih Hallit, Alvaro Postigo, Diana Malaeb, Fouad Sakr, Mariam Dabbous, Amthal Alhuwailah, Hanaa Ahmed Mohamed Shuwiekh, Sahar Obeid, Feten Fekih-Romdhane, Souheil Hallit

**Affiliations:** 1 School of Arts and Sciences, Social and Education Sciences Department, Lebanese American University, Jbeil, Lebanon; 2 School of Medicine and Medical Sciences, Holy Spirit University of Kaslik, Jounieh, P.O. Box 446, Lebanon; 3 Department of Infectious Disease, Bellevue Medical Center, Mansourieh, Lebanon; 4 Department of Infectious Disease, Notre Dame des Secours University Hospital, Postal code 3, Byblos, Lebanon; 5 Department of Psychology, University of Oviedo, Oviedo, Spain; 6 College of Pharmacy, Gulf Medical University, Ajman, United Arab Emirates; 7 School of Pharmacy, Lebanese International University, Beirut, Lebanon; 8 Department of Psychology, Kuwait University, Kuwait, Kuwait; 9 Department of Psychology, Fayoum University, Fayoum, Egypt; 10 The Tunisian Center of Early Intervention in Psychosis, Department of Psychiatry “Ibn Omrane”, Razi hospital, 2010 Manouba, Tunisia; 11 Faculty of Medicine of Tunis, Tunis El Manar University, Tunis, Tunisia; 12 Applied Science Research Center, Applied Science Private University, Amman, Jordan; 13 Department of Psychology, Badr University of Cairo, Cairo, Egypt

**BMC Psychology (2024) 12:156**

10.1186/s40359-024-01631-x

Following publication of the original article, it came to the journal’s attention that the authors’ requested addition of a further affiliation for Hanaa Ahmed Mohamed Shuwiekh (affiliation 13) had not been implemented. The article has since been corrected. The publisher thanks you for reading this erratum and apologizes for any inconvenience caused.

